# Interaction of human dendritic cell receptor DEC205/CD205 with keratins

**DOI:** 10.1016/j.jbc.2024.105699

**Published:** 2024-01-30

**Authors:** Dandan Kong, Yuanying Qian, Bowen Yu, Zhenzheng Hu, Chen Cheng, Yuanyuan Wang, Zhen Fang, Jun Yu, Song Xiang, Longxing Cao, Yongning He

**Affiliations:** 1 State Key Laboratory of Systems Medicine for Cancer, Shanghai Cancer Institute, Renji Hospital, Shanghai Jiao Tong University School of Medicine, Shanghai, China; 2 Department of Immunology, School of Basic Medical Sciences, Weifang Medical University, Weifang, China; 3 Department of Biochemistry and Molecular Biology, Key Laboratory of Immune Microenvironment and Disease, Tianjin Medical University, Tianjin, China; 4 School of Life Science, Westlake University, Hangzhou, Zhejiang, China; 5 Shanghai Key Laboratory for Cancer Systems Regulation and Clinical Translation, Shanghai, China; 6 Department of Biliary-Pancreatic Surgery, Renji Hospital, Shanghai Jiao Tong University School of Medicine, Shanghai, China

**Keywords:** DEC205, CD205, keratins, mannose receptor family, keratin binding

## Abstract

DEC205 (CD205) is one of the major endocytic receptors on dendritic cells and has been widely used as a receptor target in immune therapies. It has been shown that DEC205 can recognize dead cells through keratins in a pH-dependent manner. However, the mechanism underlying the interaction between DEC205 and keratins remains unclear. Here we determine the crystal structures of an N-terminal fragment of human DEC205 (CysR∼CTLD3). The structural data show that DEC205 shares similar overall features with the other mannose receptor family members such as the mannose receptor and Endo180, but the individual domains of DEC205 in the crystal structure exhibit distinct structural features that may lead to specific ligand binding properties of the molecule. Among them, CTLD3 of DEC205 adopts a unique fold of CTLD, which may correlate with the binding of keratins. Furthermore, we examine the interaction of DEC205 with keratins by mutagenesis and biochemical assays based on the structural information and identify an XGGGX motif on keratins that can be recognized by DEC205, thereby providing insights into the interaction between DEC205 and keratins. Overall, these findings not only improve the understanding of the diverse ligand specificities of the mannose receptor family members at the molecular level but may also give clues for the interactions of keratins with their binding partners in the corresponding pathways.

DEC205 (CD205 or Ly75) is an endocytic receptor highly expressed on the dendritic cells with antigen-presenting function (1, 2, 3) and has been widely used as a receptor target in immune therapies (4, 5). DEC205 belongs to the mannose receptor family, which also includes mannose receptor (MR) (6), PLA2R (7), Endo180 (8), and FcRY (9). MR family members are type I transmembrane proteins with a large extracellular portion, a transmembrane helix, and a short cytoplasmic region (7). Their ectodomains contain an N-terminal cysteine-rich domain (CysR), a fibronectin type II domain (FnII), and eight (ten for DEC205) C-type lectin-like domains (CTLDs) (7, 10). Previous data have shown that although the MR family members might share similar structural features (11, 12, 13, 14, 15, 16, 17), they have shown different ligand binding specificities (18). For example, the CysR domain of MR is able to bind 4-SO_4_-GalNAc (19), the FnII domains of MR and Endo180 can recognize collagen specifically (6, 20, 21, 22), but the binding of 4-SO_4_-GalNAc and collagen has not been found for DEC205 (23). Instead, DEC205 could mediate the internalization of phosphorothioated cytosine–guanosine (CpG) oligonucleotides (24), which is a widely used vaccine adjuvant (25). The functional studies of DEC205 show that it can act as a receptor for dead cells (13, 26) and may recognize dead cells through cellular keratins in a pH-dependent manner (27), as acidification usually occurs during the event of apoptosis (28, 29, 30, 31), but the mechanism of the interaction between DEC205 and keratins remains unclear. Likewise, both Endo180 and PLA2R do not show binding activities with keratins (27), suggesting that despite having similar structural features, the ligand binding properties and mechanisms of MR family members are rather diverse.

Cellular keratins are assembled into intermediate filaments (IFs), which are one of the major components of the cytoskeletal network in higher eukaryotes (32, 33) and can serve as scaffolds for maintaining the mechanical properties of cells and tissues. Keratins usually contain a central rod domain of α-helical segments separated by short linker regions. The rod is flanked by nonhelical head and tail domains with glycine-rich sequences at the amino and carboxyl termini, respectively (32, 34). Previous data have shown that keratins can be cleaved by caspases during apoptosis and generate fragments as dead cell markers (35, 36), which have been used in clinical diagnosis for some diseases (37, 38). Unfortunately, although a number of keratin-binding partners have been identified in the past decades (34), the mechanisms for the interactions are largely unknown. Current data suggest that the C-terminal tail of keratins might be a favorable region for some receptors (34), but whether it is a universal binding target needs further investigation.

Here we characterize the interaction of DEC205 with keratins by combining structural studies as well as mutagenesis and biochemical assays and identify a binding motif on keratins for DEC205, which may provide mechanistic insights into the keratin recognition by the receptors.

## Results

### Crystal structure of the CysR∼CTLD3 fragment of DEC205

**Figure 1:**
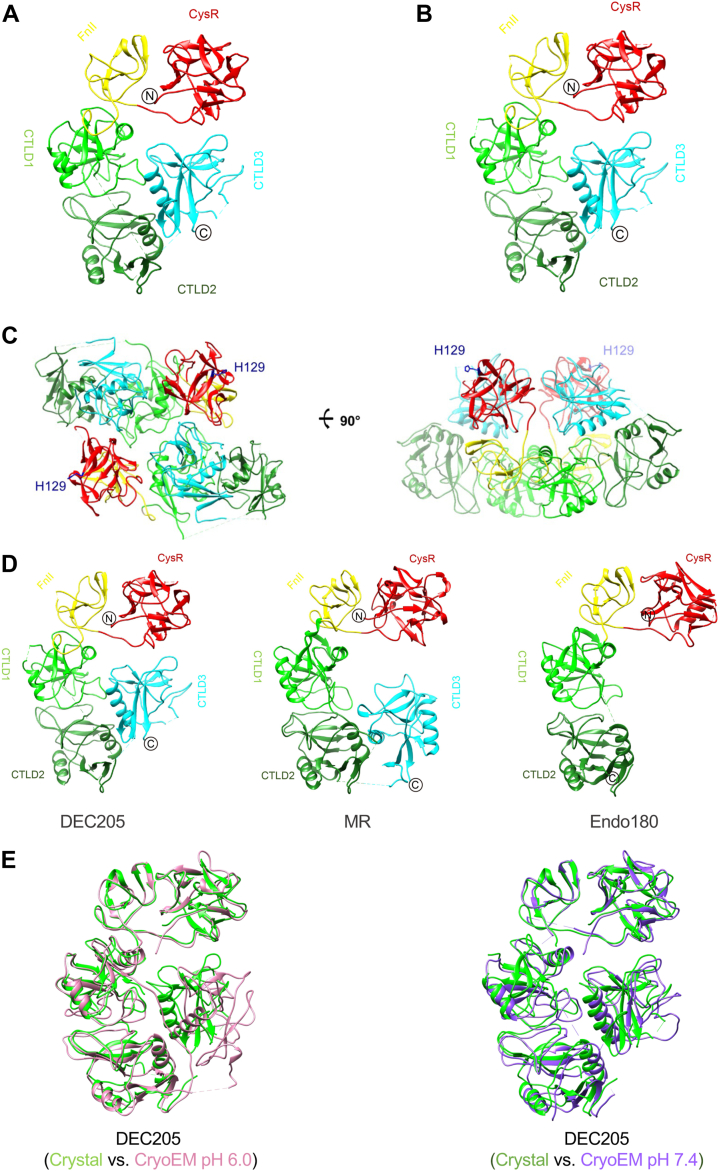
**Crystal structures of the CysR∼CTLD3 fragment of DEC205**. *A*, a ribbon diagram of the crystal structure of the CysR∼CTLD3 fragment of DEC205 in space group C2. *B*, a ribbon diagram of the crystal structure of the CysR∼CTLD3 fragment of DEC205 in space group P1. *C*, a homodimer of the CysR∼CTLD3 fragment in one asymmetric unit of P1 crystal form. CysR, FnII, CTLD1, CTLD2 and CTLD3 are colored in *red, yellow, green, forest and cyan*, respectively. The position of H129 is also shown. *D*, crystal structures of the N-terminal fragments of DEC205 (*left*), MR (*middle*, PDB: 6INN) and Endo180 (*right*, PDB: 5AO5). *E*, superposition of the crystal structure of the CysR∼CTLD3 fragment of DEC205 with the cryoEM structures determined at acidic (*left*) or basic (*right*) conditions.

Previous studies have shown that DEC205 recognizes keratin tails through its N-terminal CysR∼CTLD3 fragment at acidic pH, and CysR and CTLD3 are the two domains that might be involved in keratin recognition (13). To investigate the structure of the CysR∼CTLD3 (residues M1- S627) fragment, we expressed the fragment in insect cells and the purified protein was applied for crystallization screening. Crystals were obtained in 0.2 M Sodium Citrate with 20% PEG3350 around neutral pH (pH 6.5–7.4). X-ray diffraction data were collected and showed two crystal forms, one in space group C2 and the other in space group P1. The structures of the two crystal forms were solved by molecular replacement using the crystal structures of MR (PDB entry 6INN and 5XTS) as the search models and refined to 2.79 Å and 3.35 Å resolution, respectively (Table 1). The structures of the CysR∼CTLD3 fragment show a C-shaped conformation in both crystal forms and they are quite similar with the r.m.s. deviation of C^α^ atoms of 0.89 Å (Fig. 1, *A* and *B*). In P1 crystals, a homodimer of the fragment forms in one asymmetric unit (Fig. 1*C*), where the dimeric interface is contributed by the interactions of CysR∼CTLD1 and CTLD3 (Fig. 1*C*). The CysR∼CTLD2 region of DEC205 has an L-shaped conformation that is also found in the crystal structures of MR and Endo180 (Fig. 1*D*) (15, 39) and the C-shaped conformation of the CysR∼CTLD3 fragment of DEC205 is similar to that of MR at acidic condition (Fig. 1*D*) (17). The crystal structure of the CysR∼CTLD3 fragment is also consistent with the cryoEM structure determined recently (16), which shows that DEC205 adopts a compact lemniscate structure with two concentrated rings of CTLDs at acidic pH and can form tetramers at basic environment (16) (Fig. 1*E*). Structural superpositions show that the crystal structure has a r.m.s. deviation of C^α^ atoms of 1.5 Å with the cryoEM structure determined at the basic condition and the r.m.s. deviation of C^α^ atoms increases to 4.9 Å when superimposed with the cryoEM structure at the acidic condition (Fig. 1*E*). Within the fragment, the CysR∼CTLD2 region is relatively rigid with a r.m.s. deviations of C^α^ atoms of 1.2 Å and CTLD3 has a rotation of about 35 degrees as pH changes (Fig. 1*E*), which is analogous to the pH-dependent conformational change identified for the N-terminal fragment of MR (17). In addition, the AlphaFold model of the CysR∼CTLD3 fragment also shows that CTLD3 has an orientational change with the crystal structure (Fig. S1). The dimeric interface of the CysR∼CTLD3 fragment found in the crystals is also similar to that in the cryoEM structure determined at basic pH (16) (Fig. 1*C*), suggesting that DEC205 may dimerize on the cell surface, which might be relevant to ligand recognition.

**Table 1:** Crystallographic statistics of the structures of CysR∼CTLD3 of DEC205

Crystallographic statistics	CysR∼CTLD3 of DEC205 (PDB entry 8K8H)	CysR∼CTLD3 of DEC205 (PDB entry 8HBC)
X-ray data collection and processing		
Diffraction source	BL18U1,SSRF	BL18U1,SSRF
Wavelength (Å)	0.98	0.98
Detector	Dectris Pilatus 6M	Dectris Pilatus 6M
Temperature(K)	100	100
Space group	C 1 2 1	P 1
a, b, c (Å)	141.6 79.2 89.5	71.0 75.9 90.0
α, β, γ (°)	90 122 90	65 71 84
Resolution range(Å)	29.64–2.79 (2.86–2.79)	30.00–3.35 (3.47–3.35)
R_merge_	0.112 (0.218)	0.106 (0.243)
R_sym_	0.085 (0.221)	0.091 (0.232)
R_pim_	0.073 (0.153)	0.067 (0.159)
No. of unique reflections	21,098 (2214)	22,316 (2176)
Completeness (%)	99.41 (98.51)	97.86 (94.81)
Multiplicity	3.5 (3.0)	3.3 (3.0)
Mean I/sigma(I)	13.3 (3.47)	12.6 (3.14)
Overall B factor from Wilson plot (Å^2^)	47.25	73.92
Refinement		
Resolution range (Å)	29.64–2.79 (2.86–2.79)	29.87–3.35 (3.47–3.35)
Completeness (%)	99.41 (91.99)	97.66 (93.90)
No. of reflections, working set	20,954 (1227)	22,316 (2176)
No. of reflections, test set	1983 (129)	1998 (187)
Final R_cryst_	0.1906 (0.2608)	0.2054 (0.2737)
Final R_free_	0.2658 (0.3443)	0.2548 (0.3235)
No. of non-H atoms		
Protein	4636	9125
Solvent	67	0
Total	4703	9125
Validation		
R.m.s. deviations		
Bonds (Å)	0.009	0.003
Angles (°)	1.091	0.75
Average B factors (Å^2^)	46.0	78.43
Ramachandran plot		
Favored regions (%)	90.25	91.22
Additionally allowed (%)	8.69	8.69
Outliers (%)	0.2	0.09

Values in parentheses are for the highest resolution shells.

### The ligand-binding properties of DEC205

**Figure 2:**
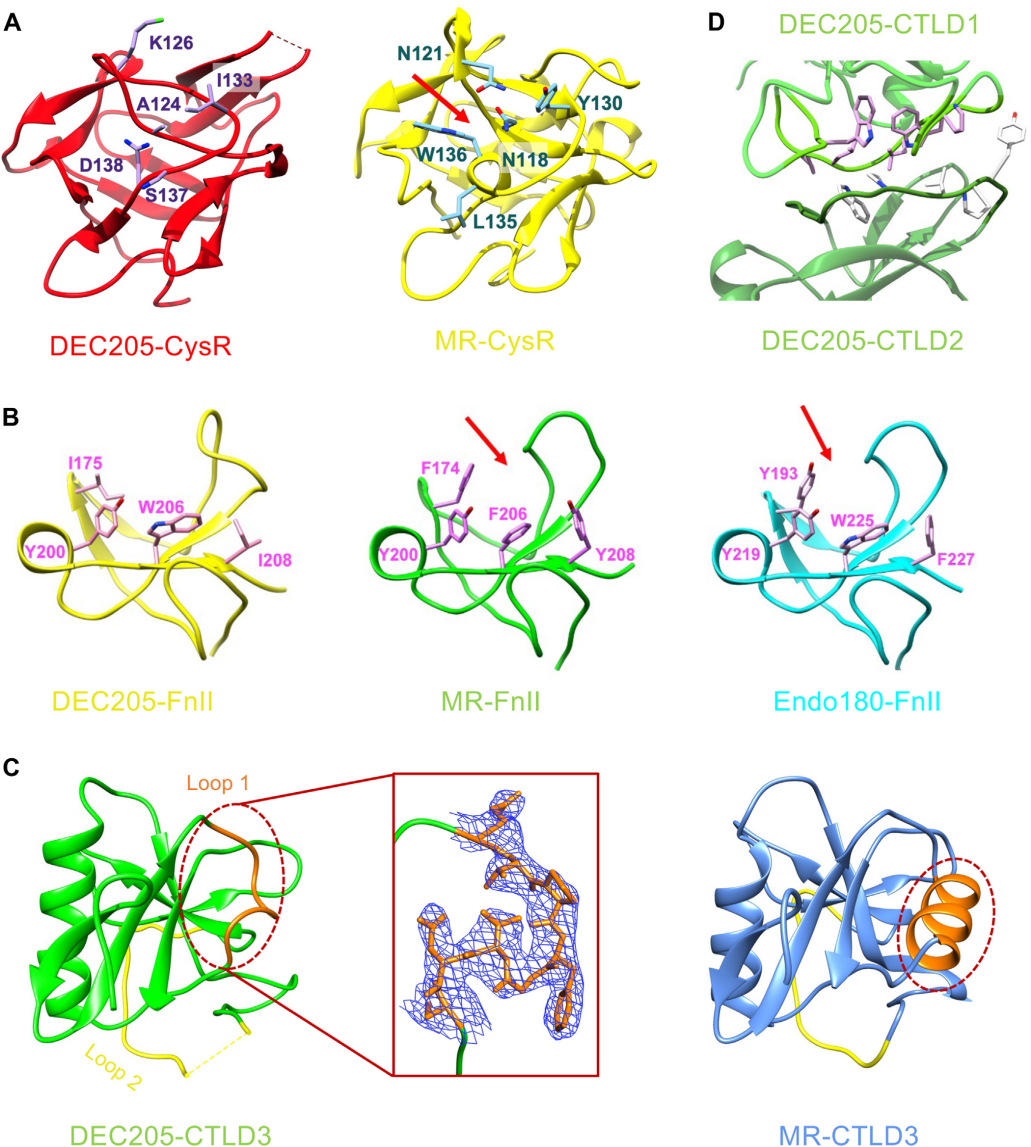
**Structural comparison of the N-terminal individual domains of DEC205 with MR and Endo180**. *A*, crystal structures of the CysR domains of DEC205 (*left, red*) and MR (*right, yellow*, PDB: 6INN). The residues around the binding pocket (*red arrow*) for 4-SO_4_-GalNAc of MR are labeled. *B*, crystal structures of the FnII domains of DEC205 (*left, yellow*), MR (*middle, green*, PDB: 6INN) and Endo180 (*right, cyan*, PDB: 5AO5). The residues around the collagen binding grooves (*red arrows*) of MR and Endo180 are labeled. *C*, the crystal structures of CTLD3 of DEC205 (*left, green*) and MR (*right, blue*, PDB: 6INN). Loop 1 (E521-C528) of CTLD3 of DEC205 and its counterpart of MR (T524-E535) are colored in *orange* (*dashed red ovals*). The electron density (*blue*) of loop 1 is shown in a *red rectangle*. Loop 2 (T573-A592) of CTLD3 of DEC205 and its counterpart of MR (E581-M593) are colored in *yellow*. The missing residues (V574-R578) in loop 2 of CTLD3 of DEC205 is shown as a *dashed yellow line*. *D*, the interface between CTLD1 (*green*) and CTLD2 (*forest*) of DEC205 in the crystal structure. The residues of CTLD1 *(purple*) and CTLD2 (*gray*) at the interface are shown.

The structure of the CysR domain of DEC205 has a trefoil shape, similar to the CysR domains of MR and Endo180 (Fig. 2*A*) (15, 39). The CysR domain of MR has a binding pocket for 4-SO_4_-GalNAc involving residues N118, N121, Y130, L135, and W136. Among them, residues L135 and W136 are located on an α-helix and form hydrogen bonds with the sulfate group of the ligand (15, 17). By contrast, the residues L135 and W136 of MR are replaced by residues S137 and D138 from a loop region of DEC205 (Fig. 2*A*), and other residues such as N118, N121, and Y130 of MR are substituted by the residues A124, K126, and I133 of DEC205 (Fig. 2*A*), thereby losing the binding activity with 4-SO_4_-GalNAc (23).

The structure of the FnII domain of DEC205 is also similar to that of MR and Endo180, consisting of two β-sheets and two conserved disulfide bonds (Fig. 2*B*). It has been shown that the FnII domains from MR and Endo180 can recognize collagen and have similar collagen-binding grooves (15, 39). However, the aromatic residues (F174 and Y208 for MR; Y193 and F227 for Endo180) that might be important for collagen recognition are replaced by I175 and I208 in DEC205 (Fig. 2*B*), respectively, which may account for the loss of collagen binding activity of DEC205 (40).

The structures of CTLD1 and CTLD2 of DEC205 exhibit typical CTLD fold (7), and the two domains are associated with each other with a hydrophobic interface as well as hydrogen bonds (Fig. 2*D*), suggesting that they might be tightly associated with each other, which is similar to MR and Endo180 (15, 39). The CTLD3 of DEC205 has a long linker with CTLD2 and, therefore, may be more flexible than CTLD1 and CTLD2, which is also analogous to MR (Fig. 1*D*) (15). Notably, the structure of the CLTD3 of DEC205 is different from the conventional CTLD fold with two unique loop regions (loop 1 and loop 2) (Fig. 2*C*). Loop 1 (residue E521-C528) replaces a conserved α-helix in the conventional CTLDs, and loop 2 (residue T573-A592) becomes much longer and partially missing in the crystal structure (Fig. 2*C*). Therefore, CTLD3 appears to be unique among the known CTLDs in the MR family. In fact, previous studies have indicated that CTLD3 might play a role in the ligand recognition of DEC205 (13, 26, 27), which may correlate with its unique structural features.

### DEC205 recognizes glycine-rich sequences on keratins

**Figure 3:**
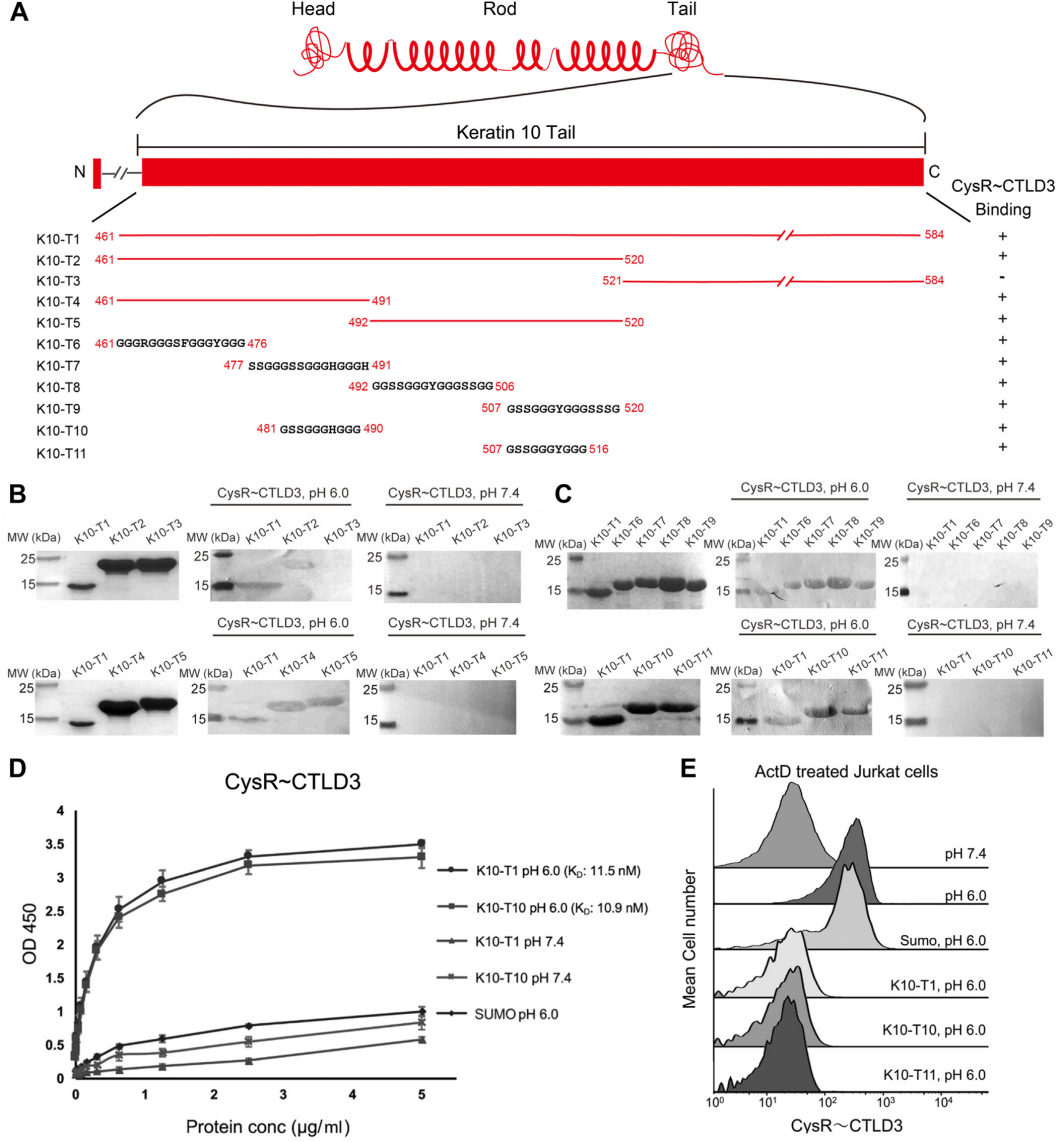
**Interactions of the N-terminal fragments of DEC205 with the K10 fragments**. *A*, schematic representation of the domain arrangement of K10 and the truncation fragments expressed for binding assays. *B*, Western blot assays of the CysR-CTLD3 fragment with the K10 fragments (T1-T5) at acidic or basic pH. The SDS-PAGE of the fragments are shown on the *left*. The Western blot results at acidic and basic pH are shown in the *middle* and on the *right*, respectively. Fragments T2-T5 are fused with a SUMO tag. *C*, Western blot assays of the CysR-CTLD3 fragment with the K10 fragments (T1, T6-T11) at acidic or basic pH. The SDS-PAGE of the fragments are shown on the *left*. The Western blot results at acidic and basic pH are shown in the *middle* and on the *right*, respectively. Fragments T6-T11 are fused with a SUMO tag. *D*, ELISA experiments of the CysR∼CTLD3 fragment with K10-T1 and K10-T10. The SUMO protein is applied as a control. *E*, inhibition of the interaction of the CysR-CTLD3 fragment with dead cells by K10-T1, K10-T10, and K10-T11 by flow cytometry. ELISA data are representative of three repeated experiments and presented as mean ± SD.

DEC205 has been shown to be able to bind the C-terminal glycine-rich region of keratins (27). To narrow down the interacting region of keratin with the N-terminal CysR∼CTLD3 fragment of DEC205, we expressed a series of truncation fragments of the C-terminal tail of keratin 10 (K10-Tn) in *E.coli* fused with a SUMO-tag (∼13 kDa) (K10-T1 was expressed without a SUMO tag), and the purified fragments were applied for Western blots with the CysR∼CTLD3 fragment of DEC205 at both acidic and basic pH. K10-T1 (residue G461-Y584) was split into K10-T2 (residue G461-G520) and K10-T3 (residue G521-Y584) (Fig. 3*A*) and only K10-T2 showed binding activity with the CysR∼CTLD3 fragment (Fig. 3*B*). K10-T2 (G461-G520) was further split into K10-T4 (residue G461-H491) and K10-T5 (residue G492-G520) (Fig. 3*A*) and both peptides had binding activities (Fig. 3*B*). Then the region containing K10-T4 and K10-T5 were divided into four fragments: K10-T6 (residue G461-G476), K10-T7 (residue S477-H491), K10-T8 (residue G492-G506) and K10-T9 (residue G507-G520) (Fig. 3*A*), and all the peptides showed binding activities with the CysR∼CTLD3 fragment (Fig. 3*C*). Considering the sequence similarities of the peptides, K10-T7 and K10-T9 were further reduced to K10-T10 (residue G481-G490) and K10-T11 (residue G507-G516) (Fig. 3*A*), respectively, and both retained the binding activities (Fig. 3*C*). Since K10-T10 and K10-T11 are quite similar with only one different residue, the ten amino acid sequence GSSGGGH/YGGG might be a motif candidate for receptor binding. The interactions of the keratin tail fragments with the CysR∼CTLD3 fragment were verified using ELISA, which showed that the K10-T1 and K10-T10 had similar affinities with DEC205 at acidic pH (Fig. 3*D*). Then, the K10-T1 fragment expressed in *E. coli* and the chemically synthesized peptides K10-T10 and K10-T11 were applied to inhibit the interaction between the CysR∼CTLD3 fragment and dead cells at acidic conditions by flow cytometry. The results showed that K10-T1, K10-T10, and K10-T11 could all block the binding between the CysR∼CTLD3 fragment and dead cells efficiently (Fig. 3*E*).

**Figure 4:**
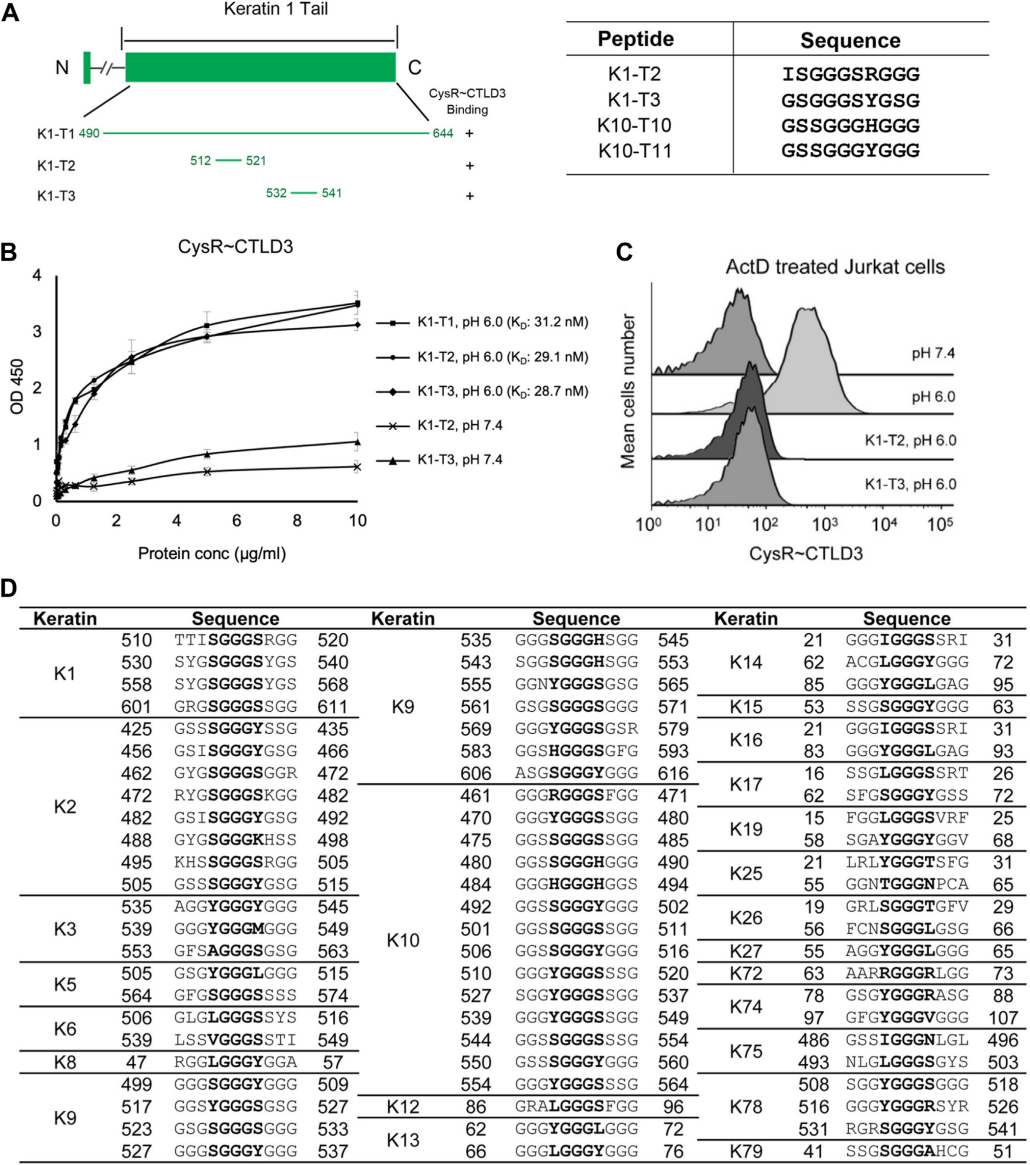
**Interactions of the N-terminal fragments of DEC205 with the K1 fragments and the motifs identified from keratins**. *A*, schematic representation of the domain arrangement of K1 and the truncation fragments expressed for binding assays (*left*) and the sequences from K1 and K10 that can interact with DEC205 (*right*). *B*, ELISA of the CysR∼CTLD3 fragment with K1-T1, K1-T2 and K1-T3. *C*, inhibition of the interaction of the CysR-CTLD3 fragment with dead cells by K1-T2 and K1-T3 by flow cytometry. *D*, the XGGGX motif identified in human keratins. The sequences of the tail or head regions of human type I and type II epithelial keratins (K1-K28, K71-K80) are downloaded from UniProt, and the XGGGX sequence are searched and picked manually. ELISA data are representative of three repeated experiments and presented as mean ± SD.

To validate the potential glycine-rich sequences identified above, we checked the tail region of keratin 1 (K1-T1, E490-R644), which had been shown to be able to bind DEC205 (27), and found that K1-T1 did not contain the binding sequences of K10 identified above, but it had similar glycine-rich sequences such as K1-T2 (I512-G521: ISGGGSRGGG) and K1-T3 (G532-G541 and G560-G569: GSGGGSYGSG) (Fig. 4*A*). Then we expressed and purified these peptides for ELISA and flow cytometry. The results showed that both K1-T2 and K1-T3 could bind the CysR∼CTLD3 fragment of DEC205 and also inhibit the interaction between the CysR∼CTLD3 fragment and dead cells (Fig. 4, *B* and *C*), suggesting that these glycine-rich sequences are important for the interactions with DEC205.

### The keratin binding motif for DEC205

**Figure 5:**
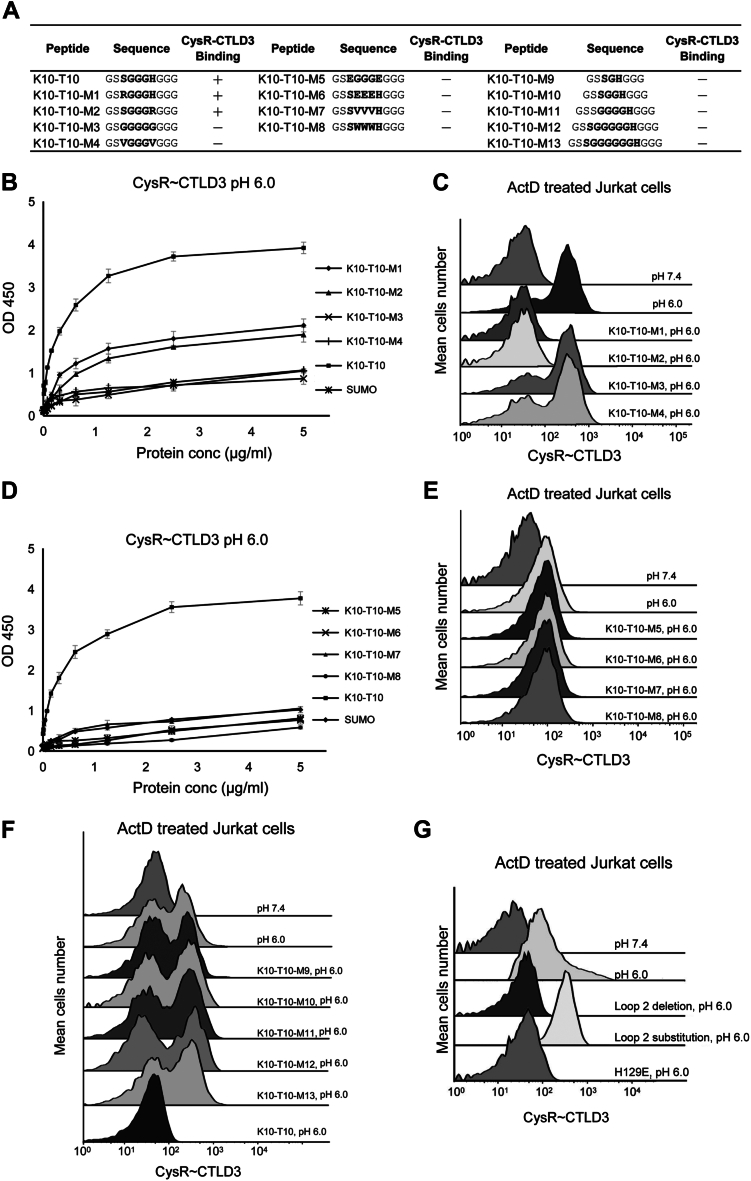
**Mutagenesis of the keratin binding motif for DEC205**. *A*, a list of mutants of the XGGGX motif applied for the binding assays with DEC205. *B*, ELISA of the CysR∼CTLD3 fragment with K10-T10-M1 to M4 at acidic pH. *C*, inhibition of the interaction of the CysR-CTLD3 fragment with dead cells by K10-T10-M1 to M4 by flow cytometry. *D*, ELISA of the CysR∼CTLD3 fragment with K10-T10-M5 to M8 at acidic pH. *E*, inhibition of the interaction of the CysR-CTLD3 fragment with dead cells by K10-T10-M5 to M8 by flow cytometry. *F*, inhibition of the interaction of the CysR-CTLD3 fragment with dead cells by K10-T10-M9 to M13 by flow cytometry. *G*, interactions of the CysR-CTLD3 fragments (wild type, deletion or substitution of a portion of loop 2 (T573-S584) of CTLD3, the H129E mutant) with dead cells by flow cytometry. ELISA data are representative of three repeated experiments and presented as mean ± SD.

According to the sequences obtained from the tails of K10 and K1 that had binding activities with DEC205, it appears that XGGGX might be a core motif of the binding peptides, where X are the non-glycine residues and preferred to be polar residues such as S, H or Y (Fig. 4*A*). Then we generated a number of point mutations (K10-T10-Mn) regarding the X residue on peptide K10-T10 for binding assays (Fig. 5*A*). ELISA data showed that mutants K10-T10-M1 and K10-T10-M2 had reduced binding activities with DEC205 when an R was introduced in the motif (Fig. 5*B*), but they still could inhibit the interaction of keratin with DEC205 (Fig. 5*C*). By contrast, when X residues were replaced by G, V, or E, the resulting peptides K10-T10-M3, K10-T10-M4 and K10-T10-M5 lost binding affinity with DEC205 (Fig. 5, *B*–*E*), suggesting that non-polar or negatively charged residues were unfavorable in the motif. Moreover, when the three glycines in the motif were substituted by E, V, and W in peptides K10-T10-M6, K10-T10-M7, and K10-T10-M8, respectively (Fig. 5*A*), the peptides showed no binding activities with DEC205, suggesting that these glycines are indispensable for the recognition (Fig. 5, *D* and *E*).

To further validate the XGGGX motif, we generated a set of peptides, K10-T10-M9 to M13, by changing the number of glycines between the X residues from one to six (Fig. 5*A*). The flow cytometry results showed that only K10-T10, which had three glycines between the X residues, could block the binding of DEC205 with dead cells, whereas other peptides that had different numbers of glycines in between revealed no inhibitory effect (Fig. 5*F*), suggesting three glycines are crucial for the interaction with DEC205.

In addition, previous mass spectrometry data showed that DEC205 could pull down a number of cellular keratins such as K1, K2, K3, K5, K6, K9, K10, K13, K14, K16, and K79, where K1, K10, K9, and K2 had higher counts in the pulldown list (27). Indeed, the sequence search results showed that the XGGGX motif can be found in the tail regions of many keratins (some are in the head regions, such as K13, K14, K16, and K79) (41), including those identified by the pull-down assays (Fig. 4*D*). And among them, K1, K10, K9, and K2 contained more copies of the motif in the tail regions (Fig. 4*D*), supporting the importance of this motif for DEC205 recognition.

### Recognition of keratins by DEC205

**Figure 6:**
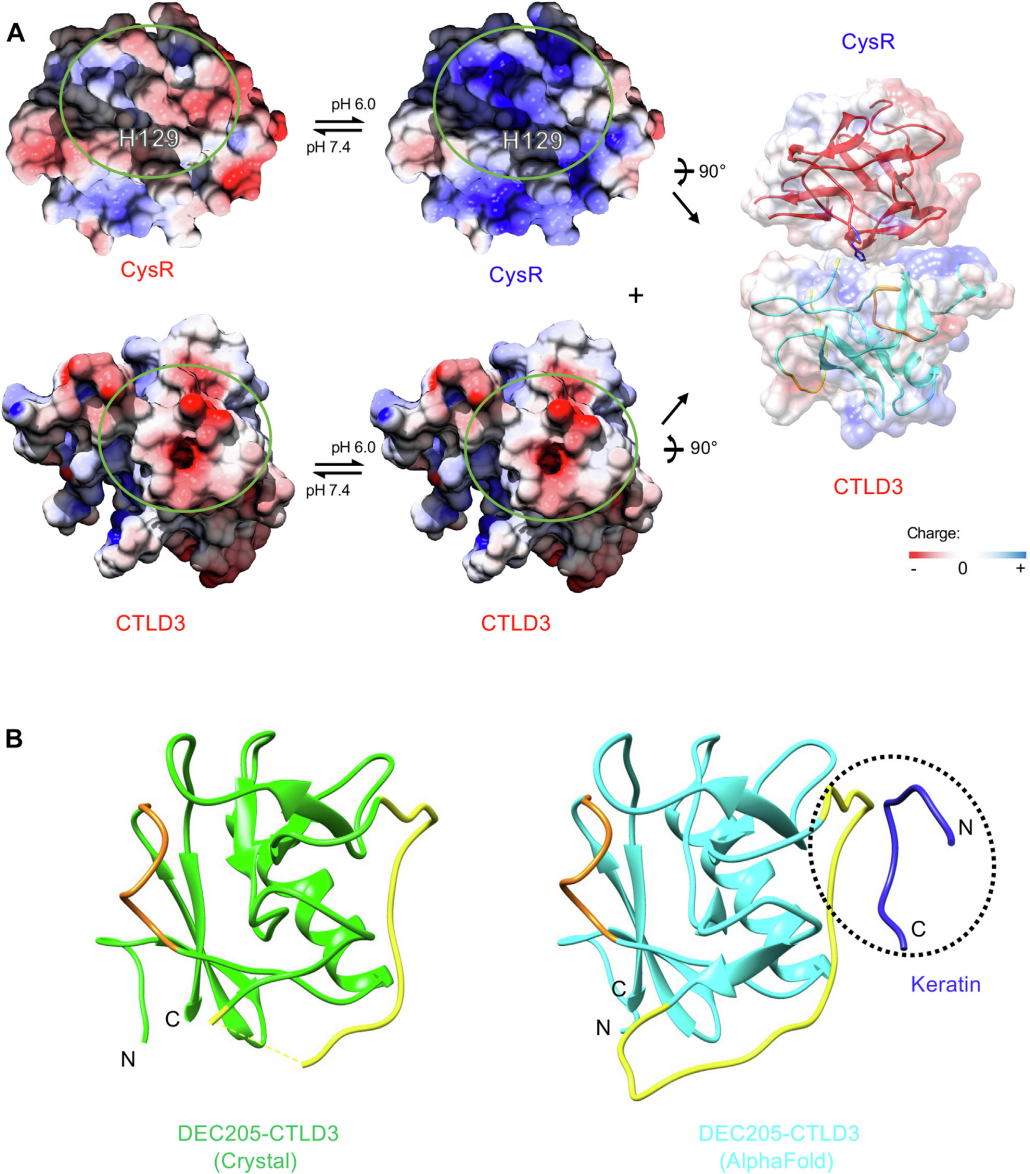
**Interaction of DEC205 with keratins**. *A*, the potential pH-dependent interaction between the CysR domain and CTLD3 of DEC205. The surface electrostatic potential of the CysR domain (*top*) and CTLD3 (*bottom*) at acidic and basic pH are shown. The positive and negative charges are colored in *blue and red*, respectively. The regions of the two domains that may contact are labeled with *green circles*. The position of H129 is also shown. *B*, the crystal structure of CTLD3 of DEC205 (*left*) and the AlphaFold prediction of the interaction of CTLD3 with a keratin peptide (K10-T10, GSSGGGHGGG, *dark blue*) (*black dashed oval; right*). Loop 1 (E521-C528) and loop 2 (T573-A592) of CTLD3 are colored in *orange* and *yellow*, respectively. The missing residues (V574-R578) in loop 2 of the crystal structure of CTLD3 is shown as a *dashed yellow line*.

Although the CysR∼CTLD3 fragment is crucial for keratin binding (13, 27), both crystal and cryoEM structures (16) do not provide obvious clues for the potential binding site for keratins. Previous data have suggested that the CysR domain and CTLD3 might interact with each other at acidic conditions, and residue H129 on the CysR domain was important for keratin recognition (Fig. 5*G*) (13, 27). However, both crystal and cryoEM structures show that H129 is pointing outward and away from CTLD3, and the intra-molecular interaction between the CysR domain and CTLD3 would be difficult due to geometric hindrance (Fig. 1*C*). Nevertheless, the interaction between the CysR domain and CTLD3 may occur in an inter-molecular fashion. To examine the possibility, we calculated the surface electrostatic potential of the CysR domain and CTLD3 at different pH (42, 43). The results showed that the surface region of CTLD3 around loop 1 and loop 2 described above (Fig. 2*C*) is negatively charged at both pH 7.4 and pH 6.0, but the surface around H129 of the CysR domain is negatively charged at pH 7.4 and becomes positively charged at pH 6.0, implying that the two regions may be able to interact with each other at acidic condition (Fig. 6*A*), as suggested by the previous biochemical results (13), and a similar mechanism has been proposed for the pH-dependent interaction of MR (17).

As described above, CTLD3 of DEC205 differs significantly from the typical CTLD fold, where loop 1 (E521-C528) replaces a conventional α-helix of CTLD and results in a much longer and flexible loop 2 (T573-A592) in the domain (Fig. 2*C*). To examine whether this unique structural feature is correlated with keratin binding, a deletion mutant of the CysR∼CTLD3 fragment, where a portion of loop 2 (T573-S584) of CTLD3 was removed, was constructed and it almost lost binding activity with dead cells (Fig. 5*G*). And when the deletion was recovered with a GS-rich sequence (SGGGSSGAGSSS), the fragment regained binding activity with dead cells (Fig. 5*G*), suggesting that loop 2 might be involved in the interaction with keratins. Furthermore, we tested the potential interaction of DEC205 with keratins by inputting the sequences of the CysR∼CTLD3 fragment and K10-T10 into AlphaFold (44, 45, 46, 47, 48), the resulting model showed that the keratin fragment K10-T10 bound predominantly on loop 2 of CTLD3 (Figs. 6*B* and S1), supporting the mutagenesis data. But how pH and the CysR domain may affect the keratin recognition as well as the binding details still need to be clarified with further evidence at molecular level.

## Discussion

Keratins are one of the major cytoskeletal components and evidence has shown that keratins could be processed during apoptosis and act as markers for dead cells (27, 36, 49), which is analogous to other cytoskeleton components such as actin or spectrin that can be recognized by the immune receptors during cell death (50, 51, 52). Although the head and tail regions have the main sequence diversity among keratins (34), the GS-rich sequences are found in the heads and tails of many keratins, and recently it has been proposed that they may be involved in phase separation and participate in multiple physiological and pathological processes (53, 54). The structural prediction by AlphaFold shows that the keratin tails are rather flexible without secondary structures (55), which may favor the interactions with DEC205 or other binding partners (34).

Previous studies have shown that keratins can bind to microbial surface components recognizing adhesive matrix molecule (MSCRAMM) called clumping factor B (ClfB), and ClfB may bind a peptide from the tail of keratin 10 with a GSSGXG motif through a dock-lock-latch model (56), where the linear peptide inserts between the N2 and the N3 domain of ClfB and form a β-sheet with N3 (56, 57). Here our evidence suggests that both CTLD3 and the CysR domain of DEC205 may be involved in recognizing keratins, and the AlphaFold prediction shows that the keratin peptide with XGGGX motif binds mainly on loop 2 of CTLD3, which is consistent with the mutagenesis data and previous results (13, 26, 27). But the role of the CysR domain and how pH may affect the binding still need further investigation. In addition, previous reports have shown that oligomerization could be a common feature for the MR family members which may correlate with ligand recognition (40, 58, 59, 60). Recent cryoEM data also suggest that DEC205 may form dimers or tetramers, which might be relevant to CpG binding (16). Similarly, the dimerization or oligomerization of DEC205 may also promote the binding of keratins.

The structure of the N-terminal fragment of DEC205 shows similar features with MR and Endo180 (15, 17, 39), suggesting the evolutionary relations among the family members; however, the structural details of the individual domains are rather different. The 4-SO_4_-GalNAc binding pocket of the CysR domain and the collagen-binding groove of the FnII domain are both modified in DEC205, therefore losing the affinities for the ligands (18, 19, 23). Early evidence has shown that CTLD3 of DEC205 could be involved in recognizing ligands during apoptosis and necrosis (26) and subsequent data suggest that it might be important for keratin binding (13, 27), which might correlate with the unique structural features of CTLD3. Therefore, the distinct structural details of the MR family members may contribute to the diverse ligand-binding properties of the receptors.

It has been shown that keratins are associated with more than 60 human disorders and could work as diagnostic markers for various diseases (37, 61). For example, overexpressed, modified, or aggregated keratins are found in several kinds of malignant cells (37), and keratins may also serve as markers for cell or tissue injury and inflammatory and immune responses (62). Therefore, as a scavenging receptor against keratins, DEC205 may have advantages in the clearance of damaged or dead cells in these cases and provide opportunities to develop biomaterials or peptide-fusing antigens based on the binding motif targeting dendritic cells for antigen delivery and presentation.

## Experimental procedures

### Protein expression and purification

For crystallization and structural determination, a construct encoding human CysR∼CTLD3 (residues M1–S627) with a C-terminal 6xHis-tag was sub-cloned into the pFastBac vectors (Invitrogen). For Western blot assays and ELISA, a human Fc homodimeric fragment with 6xHis-tag at its C-termini was fused to the C-termini of CysR∼CTLD3. In parallel, CysR∼CTLD3 fused with GFP at the C-terminus was also sub-cloned into a pFastBac vector. The Sf9 cells were used for generating recombinant baculoviruses and High-Five (Hi5) cells were used for protein production. The infected cells were cultured in ESF921 medium (Expression Systems) for 3 days in a 27 °C humidified incubator. The supernatants of the culture media were harvested and buffer-exchanged with 25 mM Tris, 150 mM NaCl at pH 8.0 by dialysis, then applied to Ni-NTA chromatography (Ni-NTA Superflow, Qiangen). The eluted proteins were further purified by gel filtration chromatography with a HiLoad Superdex 200 16/600 pg column (GE Healthcare). All DEC205 samples were prepared following similar procedures.

Human keratin K10-T1 (residue G461–Y584) and K1-T1 (residue E490-R644) fused with a C-terminal 6xHis-tag, and K10 tail fragments (T2-T11: residues G461–G520, G521–Y584, G461–H491,G492–G520, G461–G476, S477–H491, G492–G506, G507–G520, G481-G490, G507–G516) and K1 tail fragments (T2-T3: residues I512-G521, G532-G541) fused with an N-terminal 6xHis-tag followed by a SUMO tag were all expressed in *E. coli* BL21(DE3) cells (Novagen) using the pET28a expression vector and purified as soluble proteins from the supernatants of cell lysates by Ni-NTA chromatography.

### Crystallization and structural determination

The purified proteins (CysR∼CTLD3) were buffer-exchanged into 5 mM Tris, 100 mM NaCl (pH 7.4) at 10 mg/ml concentration (measured by UV absorption at 280 nm). Crystal screening was performed at 18 °C by hanging-drop vapor diffusion method using 48-well plates (Hampton Research, Molecular Dimensions, Wizard). The crystals were obtained in 0.2 M Sodium Citrate, 20% PEG3350, with pH ranging from 6.5 to pH 7.4. Diffraction data were collected using a PILATUS6M detector at BL18U beamline of the National Facility for Protein Science Shanghai (NFPS) at Shanghai Synchrotron Radiation Facility (SSRF) and processed using the HKL3000 package (63). The diffraction data statistics are summarized in Table 1. Two crystal forms are found, one in space group C2 and the other in space group P1. The structures were solved by molecular replacement using the structures of human mannose receptor (PDB: 5XTS and 6INN) as search models by PHASER (64) and refined to 2.79 Å and 3.35 Å resolution, respectively, using Coot and Phenix (65, 66). The refinement statistics are summarized in Table 1.

The surface electrostatic potential of the CysR domain and CTLD3 at different pH were calculated using PDB2PQR and PROPKA3 (42, 43). The interaction of DEC205 with keratin was predicted by giving the sequences of the CysR∼CTLD3 fragment and K10-T10 (GSSGGGHGGG) to ColabFold v1.5.3 (AlphaFold2.ipynb) based on AlphaFold2 and AlphaFold2-multimer (44, 45, 46, 47, 48, 67). Figures were generated using UCSF Chimera (68, 69).

### Western blot assays

Keratin fragments from human K1 or K10 expressed in *E. coli* were loaded onto SDS/PAGE (Bio-Rad Laboratories) and transferred onto PVDF membranes. The membranes were blocked with a blocking buffer (PBS, 5% (wt/vol) milk, 0.1% Tween 20, pH 6.0 or pH 7.4) and incubated in a blocking buffer containing the CysR∼CTLD3-Fc of DEC205 (10 μg/ml) for 2 h at room temperature. After washing three times (PBS, 0.1% Tween 20, pH 6.0 or pH 7.4), the membranes were incubated with HRP-conjugated mouse anti-human IgG Fcγ fragment-specific antibody (The Jackson Laboratory) and detected with the DAB reagent.

### ELISA experiments

Keratin fragments from human K1 or K10 were buffer exchanged with 100 mM KCl, 10 mM Tris (pH 7.0) by dialysis, and then coated onto 96-well plates with ∼2 μg protein per well at 4 °C overnight. The plates were then blocked with blocking buffers (PBS with 2% (wt/vol) BSA) with different pH values at 37 °C for 3 h. CysR∼CTLD3-Fc were serially diluted and added to each well in a binding buffer (PBS, 2 mg/ml BSA) with preset pH values. After 2 h of incubation at 37 °C, the plates were washed five times with pH-adjusted PBS (PBS, 0.1% Tween 20). The bound recombinant proteins were detected by HRP-conjugated mouse anti-human IgG Fcγ fragment-specific antibody (The Jackson Laboratory). After washing, 100 μl of chromogenic substrate (1 μg/ml tetramethylbenzidine, 0.006% H_2_O_2_ in 0.05 M phosphate citrate buffer, pH 5.0) was added to each well and incubated for 30 min at 37 °C. Then, 50 μl H_2_SO_4_ (2.0 M) was added to each well to stop the reactions. The plates were read at 450 nm on a Synergy Neo machine (BioTek Instruments). The ELISA data shown in the figures are representative of three repeated experiments and presented as mean ± SD. The K_D_ values were calculated based on the fitting of the sigmoidal curves using the software GraphPad Prism 9 (70, 71).

### Flow cytometry

Jurkat T cells were cultured in 1640 medium (Gibco) supplemented with 10% (vol/vol) FBS (HyClone Laboratories). To induce apoptosis and necrosis, Jurkat cells were incubated in tissue culture flasks for several hours with 1 μg/ml actinomycin D (ActD) until use. The ActD-induced cells were washed with PBS (pH 7.4), and then washed with either PBS at pH 7.4 or PBS at pH 6.0 for different assays. The cells were incubated with the CysR∼CTLD3-GFP fragments in PBS (pH 7.4 or 6.0) for 20 min at room temperature and then washed by PBS (pH 7.4 or pH 6) again, and analyzed by flow cytometry. For keratin inhibition assays, the cells were washed with PBS (pH 6.0) and incubated with the CysR∼CTLD3-GFP fragments with or without the keratin fragments. The concentrations of K1 or K10 fragments were about 20 μg/ml. After washing twice with PBS (pH 6.0) again, the cells were analyzed by a Becton Dickinson FACS Caliber flow cytometer (Becton Dickinson) and CELLQuest software. Data analysis was performed using FlowJo software (Tree Star, Inc).

## Data availability

The two crystal structures of the CysR∼CTLD3 fragment of human DEC205 are deposited in PDB (www.rcsb.org) with PDB entry 8HBC and 8K8H, respectively.

## Supporting information

This article contains supporting information.

## Conflict of interest

The authors declare that they have no known competing financial interests or personal relationships that could have appeared to influence the work reported in this paper.

## References

[1] Jiang W., Swiggard W.J., Heufler C., Peng M., Mirza A., Steinman R.M. (1995). The receptor DEC-205 expressed by dendritic cells and thymic epithelial cells is involved in antigen processing. Nature.

[2] Geijtenbeek T.B., van Vliet S.J., Engering A., t Hart B.A., van Kooyk Y. (2004). Self- and nonself-recognition by C-type lectins on dendritic cells. Annu. Rev. Immunol..

[3] Hawiger D., Inaba K., Dorsett Y., Guo M., Mahnke K., Rivera M. (2001). Dendritic cells induce peripheral T cell unresponsiveness under steady state conditions *in vivo*. J. Exp. Med..

[4] Bonifaz L.C., Bonnyay D.P., Charalambous A., Darguste D.I., Fujii S., Soares H. (2004). *In vivo* targeting of antigens to maturing dendritic cells *via* the DEC-205 receptor improves T cell vaccination. J. Exp. Med..

[5] Steinman R.M. (2003). The control of immunity and tolerance by dendritic cell. Pathol. Biol. (Paris).

[6] Martinez-Pomares L. (2012). The mannose receptor. J. Leukoc. Biol..

[7] East L., Isacke C.M. (2002). The mannose receptor family. Biochim. Biophys. Acta.

[8] Engelholm L.H., Ingvarsen S., Jürgensen H.J., Hillig T., Madsen D.H., Nielsen B.S. (2009). The collagen receptor uPARAP/Endo180. Front. Biosci. (Landmark Ed.).

[9] West A.P., Herr A.B., Bjorkman P.J. (2004). The chicken yolk sac IgY receptor, a functional equivalent of the mammalian MHC-related Fc receptor, is a phospholipase A2 receptor homolog. Immunity.

[10] Napper C.E., Dyson M.H., Taylor M.E. (2001). An extended conformation of the macrophage mannose receptor. J. Biol. Chem..

[11] Boskovic J., Arnold J.N., Stilion R., Gordon S., Sim R.B., Rivera-Calzada A. (2006). Structural model for the mannose receptor family uncovered by electron microscopy of Endo180 and the mannose receptor. J. Biol. Chem..

[12] He Y., Bjorkman P.J. (2011). Structure of FcRY, an avian immunoglobulin receptor related to mammalian mannose receptors, and its complex with IgY. Proc. Natl. Acad. Sci. U. S. A..

[13] Cao L., Shi X., Chang H., Zhang Q., He Y. (2015). pH-Dependent recognition of apoptotic and necrotic cells by the human dendritic cell receptor DEC205. Proc. Natl. Acad. Sci. U. S. A..

[14] Dong Y., Cao L., Tang H., Shi X., He Y. (2017). Structure of human M-type phospholipase A2 receptor revealed by cryo-electron microscopy. J. Mol. Biol..

[15] Hu Z., Shi X., Yu B., Li N., Huang Y., He Y. (2018). Structural insights into the pH-dependent conformational change and collagen recognition of the human mannose receptor. Structure.

[16] Gully B.S., Venugopal H., Fulcher A.J., Fu Z., Li J., Deuss F.A. (2021). The cryo-EM structure of the endocytic receptor DEC-205. J. Biol. Chem..

[17] Hu Z., Wang Y., Cheng C., He Y. (2019). Structural basis of the pH-dependent conformational change of the N-terminal region of human mannose receptor/CD206. J. Struct. Biol..

[18] East L., Rushton S., Taylor M.E., Isacke C.M. (2002). Characterization of sugar binding by the mannose receptor family member, Endo180. J. Biol. Chem..

[19] Fiete D., Beranek M.C., Baenziger J.U. (1997). The macrophage/endothelial cell mannose receptor cDNA encodes a protein that binds oligosaccharides terminating with SO4-4-GalNAcbeta1,4GlcNAcbeta or Man at independent sites. Proc. Natl. Acad. Sci. U. S. A..

[20] Wienke D., MacFadyen J.R., Isacke C.M. (2003). Identification and characterization of the endocytic transmembrane glycoprotein Endo180 as a novel collagen receptor. Mol. Biol. Cell.

[21] Napper C.E., Drickamer K., Taylor M.E. (2006). Collagen binding by the mannose receptor mediated through the fibronectin type II domain. Biochem. J..

[22] Madsen D.H., Leonard D., Masedunskas A., Moyer A., Jurgensen H.J., Peters D.E. (2013). M2-like macrophages are responsible for collagen degradation through a mannose receptor-mediated pathway. J. Cell Biol..

[23] Leteux C., Chai W., Loveless R.W., Yuen C.T., Uhlin-Hansen L., Combarnous Y. (2000). The cysteine-rich domain of the macrophage mannose receptor is a multispecific lectin that recognizes chondroitin sulfates A and B and sulfated oligosaccharides of blood group Lewis(a) and Lewis(x) types in addition to the sulfated N-glycans of lutropin. J. Exp. Med..

[24] Lahoud M.H., Ahmet F., Zhang J.G., Meuter S., Policheni A.N., Kitsoulis S. (2012). DEC-205 is a cell surface receptor for CpG oligonucleotides. Proc. Natl. Acad. Sci. U. S. A..

[25] Davila E., Celis E. (2000). Repeated administration of cytosine-phosphorothiolated guanine-containing oligonucleotides together with peptide/protein immunization results in enhanced CTL responses with anti-tumor activity. J. Immunol..

[26] Shrimpton R.E., Butler M., Morel A.S., Eren E., Hue S.S., Ritter M.A. (2009). CD205 (DEC-205): a recognition receptor for apoptotic and necrotic self. Mol. Immunol..

[27] Cao L., Chang H., Shi X., Peng C., He Y. (2016). Keratin mediates the recognition of apoptotic and necrotic cells through dendritic cell receptor DEC205/CD205. Proc. Natl. Acad. Sci. U. S. A..

[28] Matsuyama S., Llopis J., Deveraux Q.L., Tsien R.Y., Reed J.C. (2000). Changes in intramitochondrial and cytosolic pH: early events that modulate caspase activation during apoptosis. Nat. Cell Biol..

[29] Gottlieb R.A., Nordberg J., Skowronski E., Babior B.M. (1996). Apoptosis induced in Jurkat cells by several agents is preceded by intracellular acidification. Proc. Natl. Acad. Sci. U. S. A..

[30] Lagadic-Gossmann D., Huc L., Lecureur V. (2004). Alterations of intracellular pH homeostasis in apoptosis: origins and roles. Cell Death Differ..

[31] De Vito P. (2006). The sodium/hydrogen exchanger: a possible mediator of immunity. Cell Immunol..

[32] Jacob J.T., Coulombe P.A., Kwan R., Omary M.B. (2018). Types I and II keratin intermediate filaments. Cold Spring Harb. Perspect. Biol..

[33] Coulombe P.A., Omary M.B. (2002). 'Hard' and 'soft' principles defining the structure, function and regulation of keratin intermediate filaments. Curr. Opin. Cell Biol..

[34] Yu B., Kong D., Cheng C., Xiang D., Cao L., Liu Y. (2022). Assembly and recognition of keratins: a structural perspective. Semin. Cell Dev. Biol..

[35] Marceau N., Schutte B., Gilbert S., Loranger A., Henfling M.E., Broers J.L. (2007). Dual roles of intermediate filaments in apoptosis. Exp. Cell Res..

[36] Caulín C., Salvesen G.S., Oshima R.G. (1997). Caspase cleavage of keratin 18 and reorganization of intermediate filaments during epithelial cell apoptosis. J. Cell Biol..

[37] Toivola D.M., Boor P., Alam C., Strnad P. (2015). Keratins in health and disease. Curr. Opin. Cell Biol..

[38] Karantza V. (2011). Keratins in health and cancer: more than mere epithelial cell markers. Oncogene.

[39] Paracuellos P., Briggs D.C., Carafoli F., Lončar T., Hohenester E. (2015). Insights into collagen uptake by C-type mannose receptors from the crystal structure of Endo180 domains 1-4. Structure.

[40] Martinez-Pomares L., Wienke D., Stillion R., McKenzie E.J., Arnold J.N., Harris J. (2006). Carbohydrate-independent recognition of collagens by the macrophage mannose receptor. Eur. J. Immunol..

[41] UniProt Consortium (2022). UniProt: the universal protein knowledgebase in 2023. Nucleic Acids Res..

[42] Dolinsky T.J., Nielsen J.E., McCammon J.A., Baker N.A. (2004). PDB2PQR: an automated pipeline for the setup of Poisson-Boltzmann electrostatics calculations. Nucleic Acids Res..

[43] Olsson M.H., Sondergaard C.R., Rostkowski M., Jensen J.H. (2011). PROPKA3: consistent treatment of internal and surface residues in empirical pKa predictions. J. Chem. Theor. Comput..

[44] Eastman P., Swails J., Chodera J.D., McGibbon R.T., Zhao Y., Beauchamp K.A. (2017). OpenMM 7: rapid development of high performance algorithms for molecular dynamics. PLoS Comput. Biol..

[45] Mirdita M., Schutze K., Moriwaki Y., Heo L., Ovchinnikov S., Steinegger M. (2022). ColabFold: making protein folding accessible to all. Nat. Methods.

[46] Mirdita M., Steinegger M., Soding J. (2019). MMseqs2 desktop and local web server app for fast, interactive sequence searches. Bioinformatics.

[47] Mirdita M., von den Driesch L., Galiez C., Martin M.J., Soding J., Steinegger M. (2017). Uniclust databases of clustered and deeply annotated protein sequences and alignments. Nucleic Acids Res..

[48] Mitchell A.L., Almeida A., Beracochea M., Boland M., Burgin J., Cochrane G. (2020). MGnify: the microbiome analysis resource in 2020. Nucleic Acids Res..

[49] Ndozangue-Touriguine O., Hamelin J., Breard J. (2008). Cytoskeleton and apoptosis. Biochem. Pharmacol..

[50] Ahrens S., Zelenay S., Sancho D., Hanc P., Kjaer S., Feest C. (2012). F-actin is an evolutionarily conserved damage-associated molecular pattern recognized by DNGR-1, a receptor for dead cells. Immunity.

[51] Zhang J.G., Czabotar P.E., Policheni A.N., Caminschi I., Wan S.S., Kitsoulis S. (2012). The dendritic cell receptor Clec9A binds damaged cells *via* exposed actin filaments. Immunity.

[52] Cheng C., Hu Z., Cao L., Peng C., He Y. (2019). The scavenger receptor SCARA1 (CD204) recognizes dead cells through spectrin. J. Biol. Chem..

[53] Hughes M.P., Sawaya M.R., Boyer D.R., Goldschmidt L., Rodriguez J.A., Cascio D. (2018). Atomic structures of low-complexity protein segments reveal kinked β sheets that assemble networks. Science.

[54] Quiroz F.G., Fiore V.F., Levorse J., Polak L., Wong E., Pasolli H.A. (2020). Liquid-liquid phase separation drives skin barrier formation. Science.

[55] Tunyasuvunakool K., Adler J., Wu Z., Green T., Zielinski M., Žídek A. (2021). Highly accurate protein structure prediction for the human proteome. Nature.

[56] Xiang H., Feng Y., Wang J., Liu B., Chen Y., Liu L. (2012). Crystal structures reveal the multi-ligand binding mechanism of Staphylococcus aureus ClfB. PLoS Pathog..

[57] Ganesh V.K., Barbu E.M., Deivanayagam C.C., Le B., Anderson A.S., Matsuka Y.V. (2011). Structural and biochemical characterization of Staphylococcus aureus clumping factor B/ligand interactions. J. Biol. Chem..

[58] Lai J., Bernhard O.K., Turville S.G., Harman A.N., Wilkinson J., Cunningham A.L. (2009). Oligomerization of the macrophage mannose receptor enhances gp120-mediated binding of HIV-1. J. Biol. Chem..

[59] Su Y., Bakker T., Harris J., Tsang C., Brown G.D., Wormald M.R. (2005). Glycosylation influences the lectin activities of the macrophage mannose receptor. J. Biol. Chem..

[60] Roseman D.S., Baenziger J.U. (2000). Molecular basis of lutropin recognition by the mannose/GalNAc-4-SO4 receptor. Proc. Natl. Acad. Sci. U. S. A..

[61] Peralta Ramos M.L., Gonzalez J.A., Fabian L., Perez C.J., Villanueva M.E., Copello G.J. (2017). Sustainable and smart keratin hydrogel with pH-sensitive swelling and enhanced mechanical properties. Mater. Sci. Eng. C Mater. Biol. Appl..

[62] Ku N.O., Strnad P., Bantel H., Omary M.B. (2016). Keratins: biomarkers and modulators of apoptotic and necrotic cell death in the liver. Hepatology.

[63] Minor W., Cymborowski M., Otwinowski Z., Chruszcz M. (2006). HKL-3000: the integration of data reduction and structure solution--from diffraction images to an initial model in minutes. Acta Crystallogr. D Biol. Crystallogr..

[64] McCoy A.J., Grosse-Kunstleve R.W., Adams P.D., Winn M.D., Storoni L.C., Read R.J. (2007). Phaser crystallographic software. J. Appl. Crystallogr..

[65] Adams P.D., Afonine P.V., Bunkóczi G., Chen V.B., Davis I.W., Echols N. (2010). PHENIX: a comprehensive Python-based system for macromolecular structure solution. Acta Crystallogr. D Biol. Crystallogr..

[66] Emsley P., Cowtan K. (2004). Coot: model-building tools for molecular graphics. Acta Crystallogr. D Biol. Crystallogr..

[67] Bryant P., Pozzati G., Elofsson A. (2022). Improved prediction of protein-protein interactions using AlphaFold2. Nat. Commun..

[68] Pettersen E.F., Goddard T.D., Huang C.C., Couch G.S., Greenblatt D.M., Meng E.C. (2004). UCSF Chimera--a visualization system for exploratory research and analysis. J. Comput. Chem..

[69] Meng E.C., Goddard T.D., Pettersen E.F., Couch G.S., Pearson Z.J., Morris J.H. (2023). UCSF ChimeraX: tools for structure building and analysis. Protein Sci..

[70] Cerny L.C., Stasiw D.M., Zuk W. (1981). The logistic curve for the fitting of sigmoidal data. Physiol. Chem. Phys..

[71] Raghava G.P., Agrewala J.N. (1994). Method for determining the affinity of monoclonal antibody using non-competitive ELISA: a computer program. J. Immunoassay.

