# Supplementary material for: Interaction of human dendritic cell receptor DEC205/CD205 with keratins
Source: J Biol Chem. 2024 Jan 30;300(3):105699. doi: 10.1016/j.jbc.2024.105699 (PMC10914487; doi:10.1016/j.jbc.2024.105699)
Supplement: Figure S1 [file mmc1.docx]

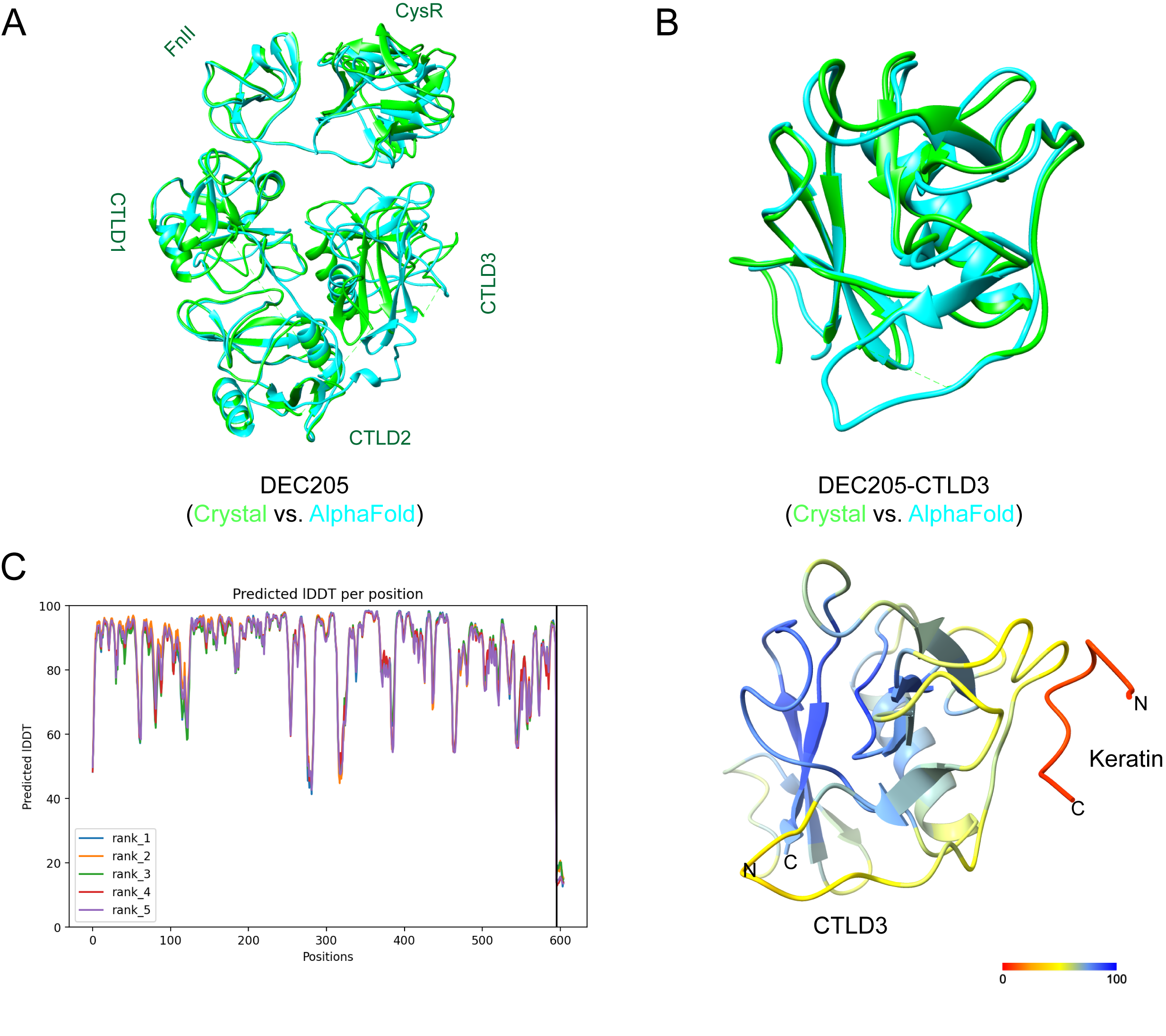


**Figure S1. The AlphaFold models of the DEC205 fragment and keratin binding**

(A) Superposition of the AlphaFold model of the CysR~CTLD3 fragment of DEC205 (cyan) with the crystal structure (green). The r.m.s. deviations of C^α^ atoms for the CysR~CTLD2 fragment and the CysR~CTLD3 fragment are 1.95 Å and 5.34 Å, respectively.

(B) Superposition of the AlphaFold model of the CTLD3 of DEC205 (cyan) with the crystal structure (green). The r.m.s. deviation of C^α^ atoms is 0.97 Å.

(C) The predicted lDDT (pLDDT) of the AlphaFold models of the CysR~CTLD3 fragment (position 1~595) bound with a keratin peptide (position 596~605; K10-T10, GSSGGGHGGG) (left). The interaction of CTLD3 with the keratin peptide from the top-ranked model (rank_1) is shown on the right and the ribbon diagram is colored based on the pLDDT scores.
